# Endovascular management of a congenital hepatic arterioportal malformation in a low resource setting

**DOI:** 10.1186/s42155-022-00314-1

**Published:** 2022-08-06

**Authors:** Poonamjeet Loyal, Ravjit Sagoo

**Affiliations:** 1 Radiology Department, Aga Khan University Hospital Nairobi, P.O. BOX 30270, Nairobi, 00100 Kenya; 2 Radiology Department, Aga Khan University Hospital Nairobi, Nairobi, Kenya

**Keywords:** Congenital hepatic arterioportal fistula, Metallic coil embolization

## Abstract

**Background:**

Arterioportal malformations, a rare type of vascular malformation, have significant associated morbidity and mortality. Management requires a carefully thought out approach by a multidisciplinary team. Low resource settings have an added challenge of limited treatment options and consumables.

**Case presentation:**

We report a case of a 14-month-old male with failure to thrive due to a congenital hepatic arterioportal fistula. He was successfully treated via an endovascular approach with metallic coil embolization.

**Conclusion:**

Hepatoportal fistula, a rare hepatic vascular malformation, has limited treatment options which can further be restricted by overall patient wellness. Minimally invasive endovascular treatment options can offer a high rate of success and reverse the morbidity associated with the disease as was seen with our case.

## Introduction

Hepatic arterioportal malformations is a communication seen directly between the portal vein and hepatic artery and can be divided into congenital and acquired. Congenital ones are a rare type of vascular malformation with one study reporting prevalence of less than 10% (Zhang et al. 2015). They may be associated with certain conditions including hereditary haemorrhagic telangiectasia, Ehlers-Danlos syndrome, and biliary atresia (Burrows et al. 2001). Acquired causes of hepatic arterioportal malformations include cirrhosis, neoplasms, trauma, and iatrogenic causes including liver biopsy, biliary surgery and gastrectomy (Davenport et al. 1999; Tanaka et al. 1991; Vauthey et al. 1997).

They are often asymptomatic but in rare instances can have debilitating symptoms that threaten survival. Some of the symptoms include portal hypertension, hepatofugal flow in the portal vein which becomes arterialized, splenomegaly, hypersplenism, variceal formation, bleeding, ascites, intestinal dysfunction including malabsorption, diarrhoea and esteatorrhea (Vauthey et al. 1997). These patients are often extremely unwell and not ideal open surgical candidates. Therefore, minimally invasive treatment presents a suitable alternative.

## Case report

**Fig. 1:**
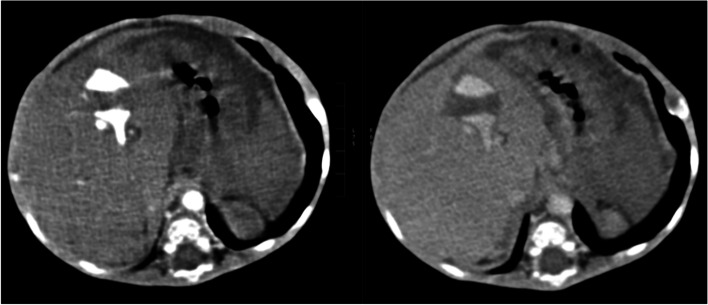
Axial CT arterial and portovenous images through the right lobe of the liver showing a tubular structure with early arterial filling and portovenous filling

**Fig. 2:**
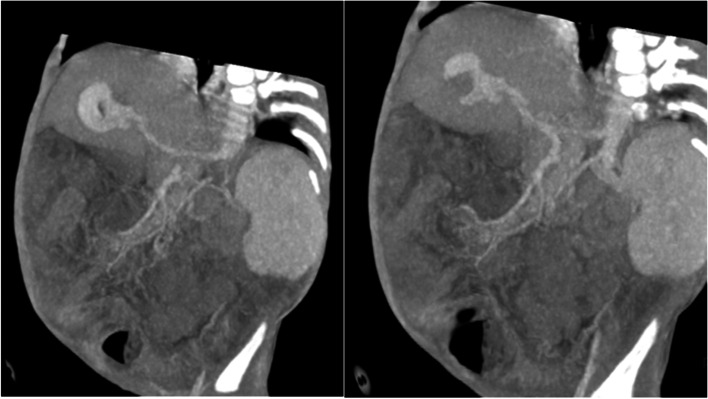
Coronal Maximum Intensity Projection images showing the high flow fistula with arterial supply from the left hepatic artery (left) and venous drainage into the left portal vein (right)

We present a case of a 14-month-old male who was referred to our facility with complete failure to thrive since birth and had developed melena stools intermittently for 1 month. The haemoglobin at presentation was 7.0 g/dl. The patient was seen at a peripheral facility but the referral to a specialist was delayed. An ultrasound and contrast-enhanced CT of the abdomen was performed which showed a 2.4 cm × 2.2 cm tubular structure in the right hepatic lobe with early arterial filling from the left hepatic artery and draining into left portal vein (Figs. 1 and 2). Early high flow retrograde arterial contrast filling of the left portal vein, the portal vein and the superior mesenteric vein, splenic vein and their tributaries was also seen. The portal vein, left portal vein, common hepatic and left hepatic artery were prominent. No other liver lesion was seen. There were also features of portal hypertension with periportal collateral vessels.

A multidisciplinary meeting was held to discuss the treatment options between the paediatrician, paediatric surgeon and interventional radiologist. The child was a poor surgical candidate as he was severely malnourished with a fairly central lesion in the right lobe of the liver. Therefore, a decision was made to attempt to treat at least the arterial inflow via an endovascular approach.

**Fig. 3:**
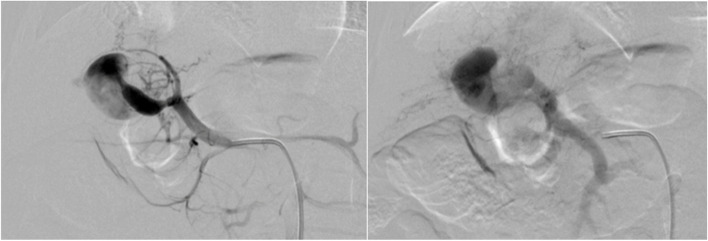
Digital subtraction angiography images demonstrating the high flow hepatic fistula with arterial supply from the left hepatic artery and venous drainage into the left portal vein

The procedure was performed under general anaesthesia. Under ultrasound guidance, a 4Fr 7 cm hydrophilic sheath (Angiogate TransRadial Introducer Kit by Kimal, United Kingdom) was placed retrogradely into the right common femoral artery. Intraarterial hourly heparin bolus was administered to prevent thrombosis at the access site and lower limb. Access to the celiac axis and then common hepatic artery was obtained using hydrophilic standard angled guide wire (Merit Laureate by MeritMedical, Ireland) and 4 Fr Cobra 2 catheter (Performa Angiographic catheter by MeritMedical, Mexico). The left intrahepatic arterioportal malformation was demonstrated receiving blood supply from the left hepatic artery and draining into left portal venous vein (Fig. 3).

**Fig. 4:**
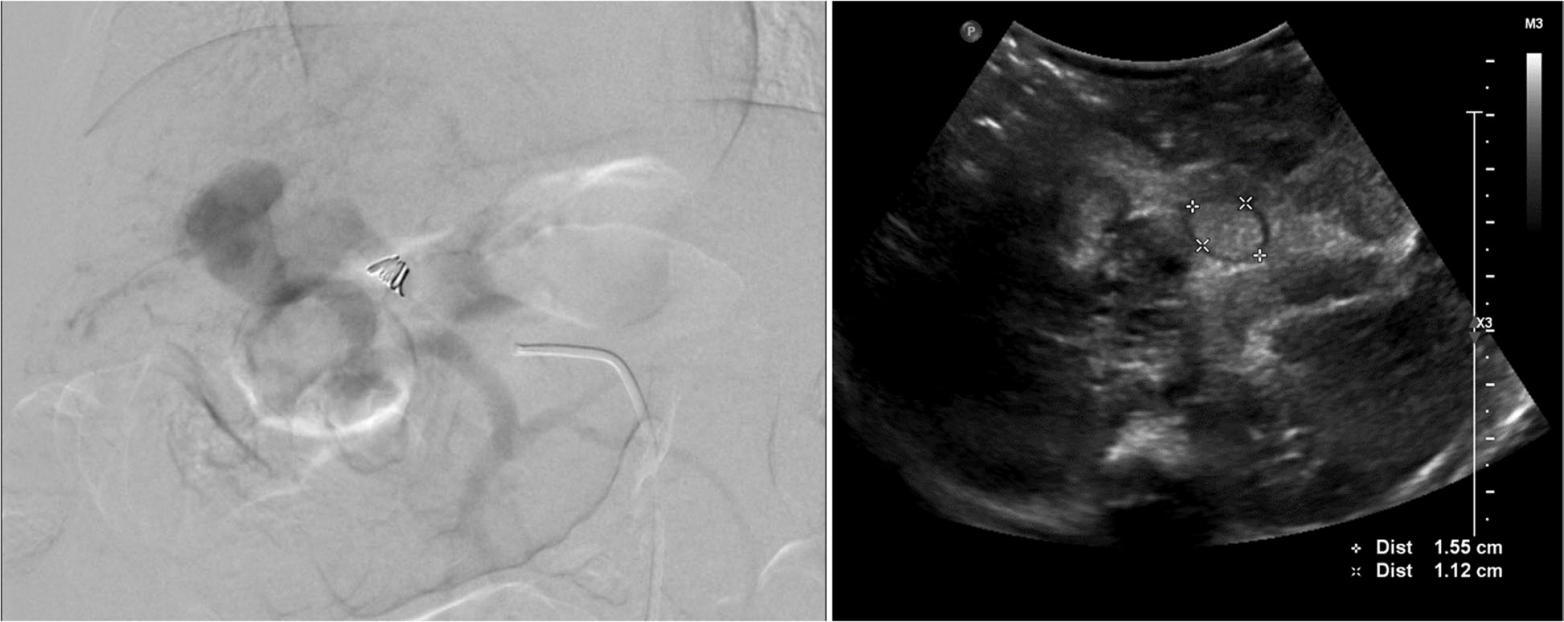
Endpoint achieved with metallic coil inserted showed no flow (left) and post procedure ultrasound showed intramural thrombus (right)

A decision was made to perform coil embolization of the main feeder as distally possible using a 2.4Fr microcatheter (Progreat by Terumo) to deploy a 4 mm detachable micro-coils (Concerto by Medtronic, The Netherlands). There was some flow still noted and hence a second 5 mm detachable micro-coil (Concerto by Medtronic, The Netherlands) was put in place which stopped the flow (Fig. 4).

There was good outcome with one tiny feeder opening immediately post embolisation. Digital subtraction angiography of the superior mesenteric artery showed no additional supply to the fistula.

An ultrasound scan performed 1 week after the procedure confirmed thrombosis of the fistula with antegrade flow in the portal vein (Fig. 4). This was maintained on further follow up scans.

A further hospital admission 2 months after the procedure was necessary to manage refeeding syndrome. However, the patient did well clinically for a number of months once discharge, gaining significant weight and no further melaena. Unfortunately, he succumbed 8 months after the procedure secondary to an unrelated, acute respiratory illness.

## Discussion

Vascular malformations are classified into fast flow, slow flow, and combined forms according to the Mullicken and Glowacki classification. Fast flow vascular malformations include the arteriovenous malformations and arterioportal fistulas. Slow flow includes portosystemic shunts, venous and lymphatic malformations. In our case, this was a fast flow arterioportal fistula. They can also be subclassified depending on symptomatology into Type 1 as small peripheral asymptomatic and like our case which is a Type 2- large central fistula causing physiological insult (Guzman et al. 2006).

Although rare, there have been cases similar to ours that have been reported. The initial modality of imaging is doppler ultrasound where there is enlargement of the hepatic artery and dilatation of the segment of the portal vein where the fistula is located (Mora et al. 1992). Pulsatile hepatofugal flow in the portal vein and color speckling in the hepatic parenchyma adjacent to the fistula may also be seen (Mallant et al. 2007).

The endpoint for treatment is restoration of normal flow dynamics in the hepatic artery and portal venous system. Treatment options are often multi-staged including embolization of the feeding artery especially if a single one is identified with possible subsequent surgery involving hepatic artery ligation/resection of lesion especially if new feeders open as a result of embolization (Akpek et al. 2001). For those patients who are unable to control symptoms, liver transplantation can be considered if available in the healthcare setting. Previously, hepatic artery ligation was considered the sole intervention, but now interventional radiology has the advantage of offering minimally invasive technique which further translates to reduced mortality and morbidity with shorter in patient stay and fewer complications. The downside of the interventional-guided embolization is that it can be limited by variant anatomy, tortuosity of vessels and elongation of the vessels die to a high dynamic flow (Oguslu et al. 2019). The access can be transfemoral and in cases where this fails, a transhepatic approach can be attempted. The latter is often fraught with more complications including peritoneal hemorrhage, fistulization, pneumothorax (Oguslu et al. 2019).

The choice of embolization agent depends on the type of arterioportal malformation, size and number of the feeding arteries, location of the fistula which further determines the accessibility (Oguslu et al. 2019). One of the main factors especially in developing countries, is availability as in our case where we used the metallic coils which is the most commonly used agent. Other options include Amplatzer plug, *N*-butyl cyanoacrylate, polyvinyl alcohol (PVA) or microsphere (Tasar et al. 2005). The Amplatzer plug is also popular as it can be used solely for occlusion of large vessels (Oguslu et al. 2019). PVA particles in general are not the preferred embolic agent as there is a large risk of non-target embolization into the venous system in high flow malformations. One study has reported successful embolization of larger arteriorportal malformations with detachable balloons which may be used either alone or in combination with other embolization agents (Tasar et al. 2005).

One of the complications to consider include migration of the embolization agent past the site of the arterioportal malformation and into a distal tributary of the portal vein which may cause portal venous thrombosis and subsequent infarction. The risk for migration of embolization increases with increasing size of the arterioportal malformation and high flow rates (Oguslu et al. 2019).

The prognosis if left untreated results in arterialization of the portal vein with early onset of portal hypertension, hepatoportal sclerosis and fibrosis of the portal radicles further contributing to portal hypertension and fatal hepatic necrosis.

## Conclusion

Hepatoportal fistula, a rare hepatic vascular malformation, has limited treatment options which can further be restricted by overall patient wellness. Minimally invasive endovascular treatment options can offer a high rate of success and reverse the morbidity associated with the disease as was seen with our case.

## Data Availability

Availability of data and materials was sought from the records office- Aga Khan University Hospital.

## Abbreviations

PVA: Polyvinyl Alcohol
CT: Computer Tomography
